# Seroprevalence and potential risk factors of peste des petits ruminants in goats in Mandhera District, Sahil Region, Somaliland

**DOI:** 10.1186/s12917-026-05359-1

**Published:** 2026-02-27

**Authors:** Abdirahman Saed Abdi, Ephrem Tora Toma

**Affiliations:** 1 Department of Planning and Research, Ministry of Livestock and Rural Development, Hargeisa, Somaliland; 2 Department of Veterinary Science, Arba Minch University, Arba Minch, Ethiopia

**Keywords:** c-ELISA, Goats, Mandhera, Risk factors, Seroprevalence, Somaliland, PPRV

## Abstract

**Background:**

Peste des Petits Ruminants (PPR) is a highly contagious viral disease of small ruminants that causes significant socio-economic losses globally. In Somaliland, while the disease is known to be endemic, data regarding its status in specific regions like the Sahil region remain fragmented. This study aimed to estimate the seroprevalence and identify potential risk factors of Peste des Petits Ruminants virus (PPRV) in unvaccinated goats in the Mandhera district, Sahil region.

**Methods:**

A cross-sectional study was conducted from September 2023 to February 2024. A multi-stage random sampling approach was used to select 522 unvaccinated goats older than 6 months. Serum samples were analyzed using competitive Enzyme-Linked Immunosorbent Assay (c-ELISA). Epidemiological data were collected via semi-structured questionnaires. Risk factors were identified using univariable and multivariable logistic regression.

**Results:**

The overall animal-level seroprevalence was 5.3% (95% CI: 3.0–8.0%). Multivariable analysis revealed that goats from medium-sized flocks (45–100 animals) were significantly more likely to be seropositive (AOR = 5.2, 95% CI: 1.5–14.3, *p* = 0.003) than those from small flocks. Co-rearing goats with sheep also increased the likelihood of seropositivity (AOR = 4.2, 95% CI: 1.7–15.7, *p* = 0.047). Conversely, goats in mixed grasslands and older age groups (> 1 year) showed lower odds of seropositivity compared to their respective counterparts.

**Conclusion:**

The findings confirm the circulation of PPRV in the Mandhera district. The association of seropositivity with flock size and mixed-species rearing suggests that management practices play a critical role in virus maintenance. Implementation of targeted vaccination and enhanced surveillance are recommended for the Sahil region.

**Supplementary Information:**

The online version contains supplementary material available at 10.1186/s12917-026-05359-1.

## Introduction

Small ruminants are vital for food security and the livelihoods of agro-pastoralists in Somaliland due to their adaptability and high reproductive rates [1]. However, the productivity of this sector is frequently hindered by environmental shocks and infectious diseases [2, 3]. Among these, Peste des Petits Ruminants (PPR), or “goat plague,” is a major constraint. Caused by *Small ruminant morbillivirus* (formerly PPRV), the disease is characterized by high morbidity and mortality, leading to direct economic losses and international trade restrictions [4, 5].

PPRV belongs to the family *Paramyxoviridae* and exists as a single serotype with four distinct lineages [6]. In the Horn of Africa, the disease is endemic, with outbreaks frequently documented in Somaliland, Somalia, Ethiopia, and Kenya [7]. The livestock sector is the backbone of the Somaliland economy, accounting for over 60% of the population’s income. Markets such as Burao and Berbera are central hubs for exporting millions of sheep and goats to the Arabian Gulf [8]. Despite this importance, trade embargos are often imposed due to concerns regarding transboundary diseases like PPR [9].

While a nationwide vaccination campaign was initiated in Somalia in 2012 to align with the FAO/WOAH global eradication strategy, surveillance remains inconsistent [10]. Previous studies in the Somali region have reported varying seroprevalence rates, ranging from 6.5% in Somaliland to over 30% in southern Somalia [10, 11]. However, there is a lack of recent, localized data for the Sahil region, specifically the Mandhera district, which serves as a critical transit zone for livestock moving toward the Berbera port.

Accurate data on seroprevalence and risk factors are essential for progressing through the stages of the PPR Global Eradication Programme (GEP). Therefore, this study was designed to estimate the seroprevalence of PPRV and identify associated risk factors in unvaccinated goats in the Mandhera district to provide a baseline for regional control strategies.

## Materials and methods

### Study area

**Fig. 1:**
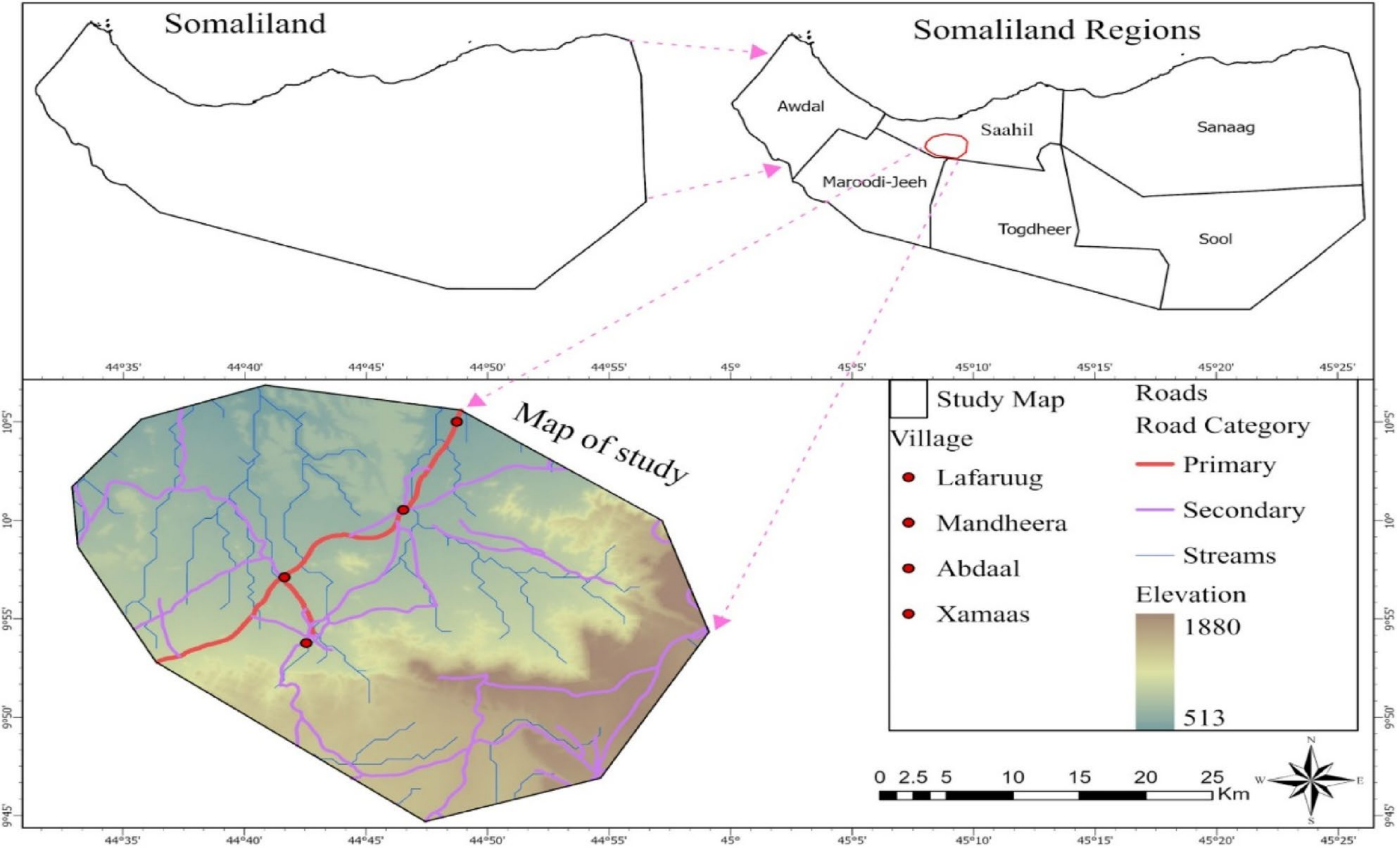
Map of the study area, (Source: Primary)

The study was conducted in Mandhera district, Sahil region (Fig. 1), which is located approximately 110 km northeast of Hargeisa. The region is characterized by a mountainous semi-desert landscape with an average annual rainfall of 200 mm and temperatures reaching 40 °C. Livestock production is predominantly pastoral and agro-pastoral [12].

### Study population and design

A cross-sectional study was conducted from September 2023 to February 2024, and targeted goats aged ≥ 6 months with no prior history of PPR vaccination. Vaccination status was verified through district records and interviews with owners and community animal health workers (CAHWs). Goats under 6 months were excluded to avoid the detection of maternal antibodies [4].

### Sampling procedure

A multi-stage random sampling approach was employed. Four villages (Mandhera, Lafaruug, Xamaas, and Abdaal) were randomly selected. Within these, 40 clusters (herds) were identified. The sample size (*n*=522*n* = 522) was calculated using the formula for two-stage cluster sampling [13], assuming an expected seroprevalence of 6.5% [10], 5% precision, and a between-cluster variance of 0.0157.

$$\mathrm{n}=\:\frac{{1.96}^{2}*g\left\{Pexp\left(1-Pexp\right)-Vc\right\}}{g*{d}^{2}-{1.96}^{2}*Vc}$$

Where, n = required sample size, g = number of clusters to be sampled = 40, Pexp = expected prevalence, d = absolute precision, Vc = between-cluster variance. **(**Table 1**).**

**Table 1:** Distribution of sample size in villages per clusters

s/*n*	Village	Number of clusters per village	Number of animals per cluster	Number of animals per village
1.	Laforuug	11	13	11*13 = 143+1 = 144
2.	Mandhera	9	13	9*13 = 117
3.	Xamaas	9	13	9*13 = 117
4.	Abdaal	11	13	11*13 = 143 + 1 = 144
	**Total**	**40**		**522**

### Sample collection and laboratory analysis

Blood (5 ml) was collected via jugular venipuncture into plain vacutainer tubes. Serum was separated by leaving blood to sediment overnight and stored at -20 °C. Serum samples were tested at the National Veterinary Laboratory Center (NVLC) in Hargeisa using a competitive ELISA (cELISA) kit (ID Screen^®^ PPR Competition, ID.vet, Grabels, France) to detect antibodies against the PPR virus nucleoprotein. Briefly, samples and controls were added to the pre-coated plates and incubated. After washing, a conjugate was added, followed by a second incubation and wash cycle. Substrate solution was then added to develop the color reaction, which was stopped with a stop solution. The optical density (OD) was read at 450 nm using a [BioTek ELx800] spectrophotometer. The results were interpreted by calculating the competition percentage (S/N% = OD sample / OD negative control × 100). As per the manufacturer’s instructions, samples with an S/N% ≤ 50% were considered positive.

### Questionnaire survey

A semi-structured questionnaire was administered to 522 goat owners to collect data on risk factors, including age, sex, flock size, grazing practices, and introduction of new animals (Additional File 1).

### Statistical analysis

Data were analyzed using STATA version 14.2 and seroprevalence calculated as the proportion of positive samples. Univariable logistic regression was used to screen variables (*p* < 0.25), followed by multivariable logistic regression to identify independent risk factors. Model fitness was assessed using the Hosmer-Lemeshow test. Statistical significance was set at *p* < 0.05.

## Results

### Seroprevalence of PPR

Of the 522 goats tested, 28 were seropositive, resulting in an overall animal-level seroprevalence of 5.3% (95% CI: 3.0–8.0%). Village-level distribution showed the highest prevalence in Lafaruug (1.7%) and the lowest in Xamaas (1.0%) (Table 2).

**Table 2:** Descriptive characteristics of study area and goats at Mandhera district of Sahil region, Somaliland, 2023 (*n* = 522)

Variables	Category	Number. Examined	Number. Seropositive	Proportion in (*n* = 522)
*Villages*	Lafaruug	144	9	1.7%
	Abdaal	144	8	1.5%
	Mandhera	117	6	1.1%
	Xamaas	117	5	1.0%
	**Total**	**522**	**28**	**5.3%**
*Agro-ecology*	Lowland	405	22	4.2%
	Midland	117	6	1.1%
	**Total**	**522**	**28**	**5.3%**
*Sex*	Male	81	5	0.9%
	Female	441	23	4.4%
	**Total**	**522**	**28**	**5.3%**
*Age*	> 0.5 < 1year	50	7	1.3%
	1-3years	306	14	2.7%
	> 3years	166	7	1.3%
	**Total**	**522**	**28**	**5.3%**

**Fig. 2:**
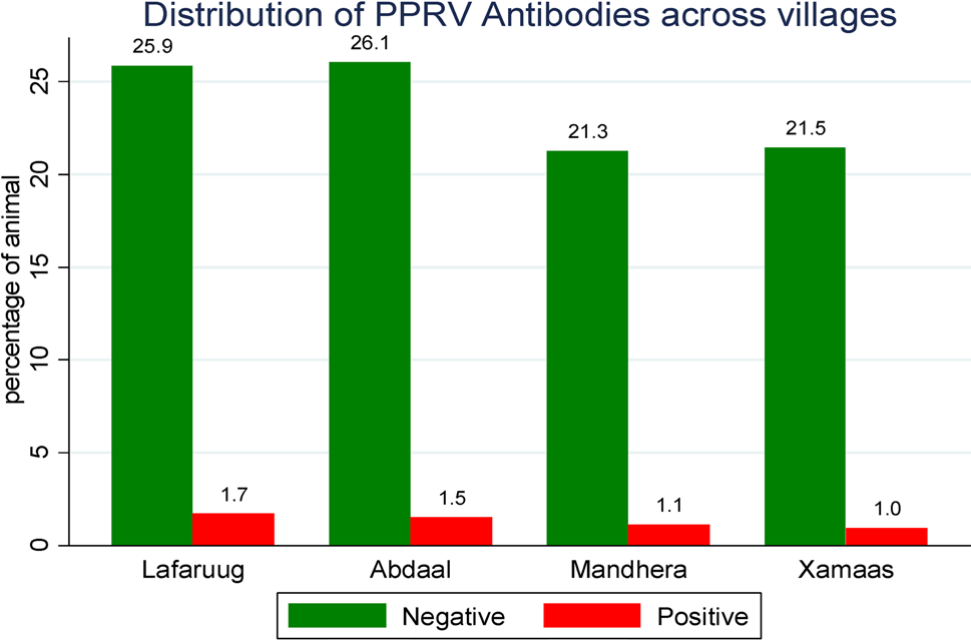
Distribution of PPRV antibodies across villages

**Fig. 3:**
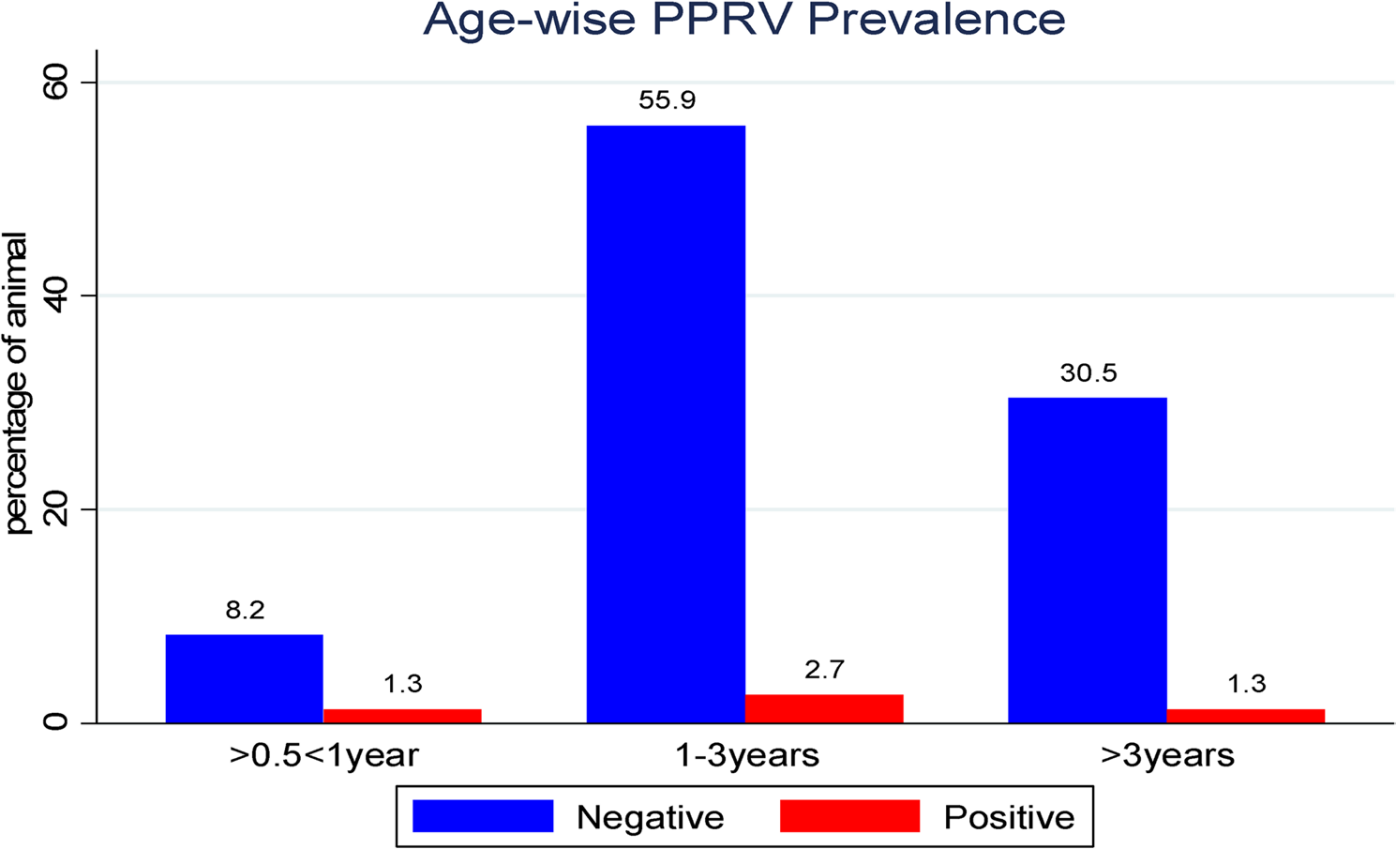
Age-specific of PPRV seroprevalence

**Fig. 4:**
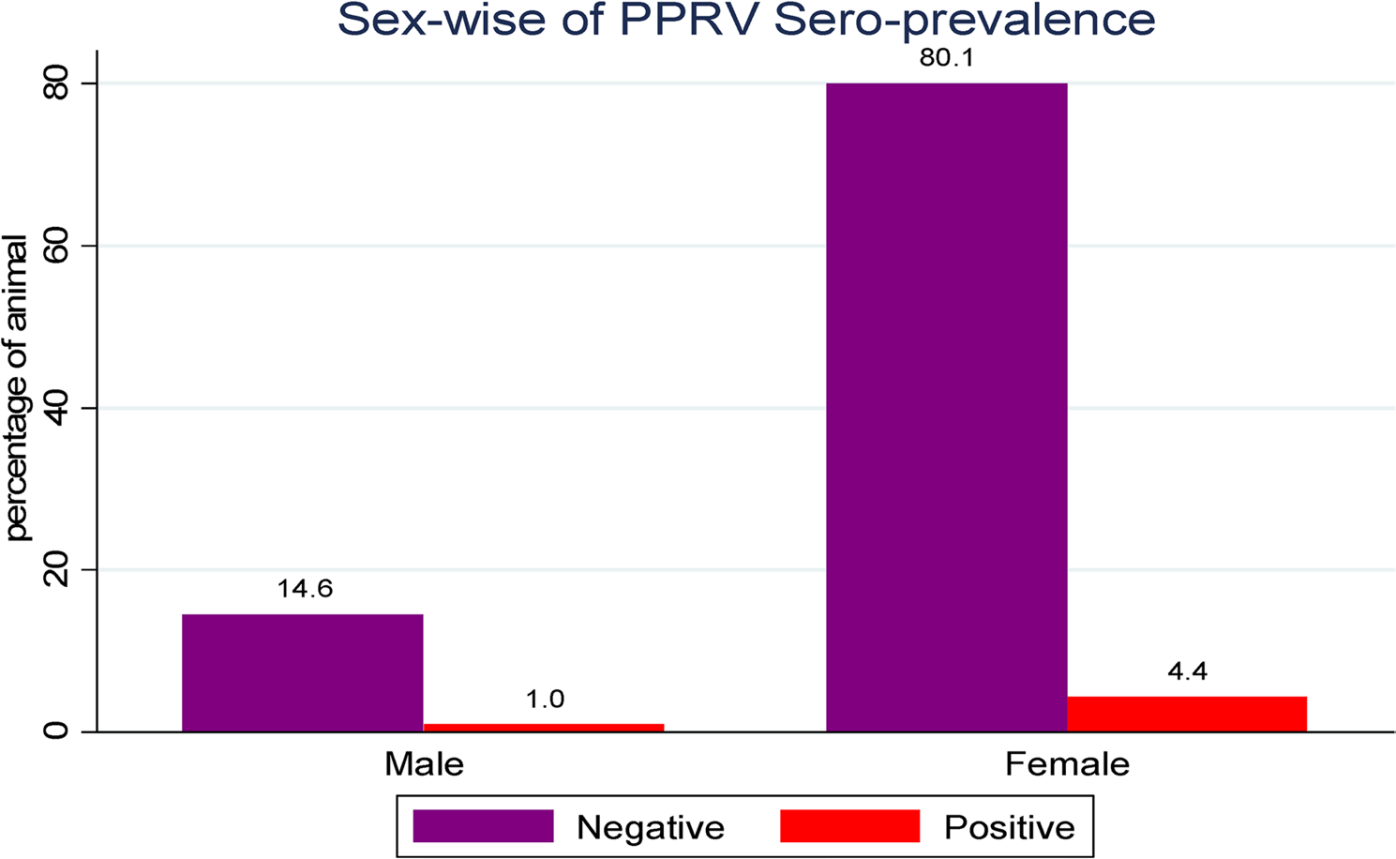
Across Sex of PPRV seroprevalence (%) in goats

At village-level, Lafaruug showed a relatively higher PPRV seroprevalence of 1.7%, compared to Abdaal (1.5%), Mandhera (1.1%), and Xamaas (1.0%), as illustrated in (Fig. 2***)***. As presented in Fig. 3, an Stratified by age comparison revealed a seroprevalence of 1.3% in goats aged between > 6 months and < 1 year, 2.7% in the 1–3 years age group, and 1.3% in those older than 3 years (Fig. 3***)***. The majority (77.6%) of the study animals were from lowland agro-ecology, with a PPRV seroprevalence of 4.2%, while midland areas accounted for 22.4% of animals and a 1.1% PPRV seroprevalence. About 84.5% of the study animals were female, showing a seroprevalence of 4.4%, whereas 15.5% were male, with a seroprevalence of 0.9%. The sex-specific seroprevalence is shown in (Fig. 4***).***

### Association of PPRV Seroprevalence with potential risk factors

#### Univariable logistic regression analysis

In the univariable logistic regression analysis, flock size and age were significantly associated with PPRV seropositivity (*P* < 0.05), Variables with *P* > 0.25 were excluded **(**Table 3**)**.

**Table 3:** Univariable logistic regression analysis of Animal-Level Seroprevalence of PPRV antibodies and risk factors in goat at Mandhera district of Sahil region, Somaliland, 2023 (*n* = 522)

Variables	Category	Number. Examined	Number. seropositive	COR	95% CI	*P*-value
*Agro-ecology*	Lowland	405	22	1.0	0.4–2.6	0.8
	Midland	117	6	Ref	-	-
*Age*	> 0.5 ≤ 1year	50	7	Ref	-	-
	1-3years	306	14	0.2	0.1–0.7	0.013*
	> 3years	166	7	0.2	0.08–0.81	0.020*
*Sex*	Male	81	5	Ref	-	-
	Female	441	23	0.8	0.3–2.2	0.726
*Flock size*	Small(< 45)	117	9	Ref	-	-
	Medium45-100	156	3	1.4	0.52–4.16	0.234
	Large > 100	249	16	5.3	1.12–14.3	0.033*
*Raising with sheep*	No	78	6	Ref	-	-
	Yes	444	22	1.6	0.24–1.59	0.327
*New animal introduction*	No Yes	391 131	18 10	Ref 1.7	- 0.7–3.8	- 0.188
*Body condition score*	Poor	38	2	Ref	-	-
	Moderate	247	14	1.0	0.2–4.9	0.920
	Good	237	12	0.9	0.2–4.4	0.959
*Animal source*	Born in	404	19	Ref	-	-
	Purchased	118	9	1.6	0.7–3.8	0.220
*Free movement*	No	79	6	Ref	-	-
	Yes	443	22	0.6	0.2–1.6	0.344
*Wildlife contact*	No	65	4	Ref	-	-
	Yes	457	24	0.8	0.2–2.5	0.763
*Housing system*	Fence stable	78	5	Ref	-	-
	House barn	53	2	0.5	0.1-3.0	0.515
	Other	391	21	0.8	0.3–2.2	0.715
*Share common house…*	No	53	5	Ref	-	-
	Yes	469	23	0.4	0.1–1.3	0.174
*Production system*	Sedentary	80	5	Ref	-	-
	Agro-pastorals	78	5	1.0	0.2–3.7	0.967
	Pastorals	364	18	0.7	0.2–2.1	0.635
*Management system*	Semi-intensive	104	7	Ref	-	-
	Extensive	418	21	0.7	0.3–1.7	0.491
*Shifting animals…*	No	79	7	Ref	-	-
	Yes	443	21	0.5	0.2–1.2	0.141
*Share common grazing land*	No	104	6	Ref	-	-
	Yes	418	22	0.9	0.3–2.3	0.838
*Grazing place*	Plain	105	9	Ref	-	-
	Mixed	78	5	0.7	0.2–2.2	0.588
	Mountainous	339	14	0.4	0.1-1.0	0.079
*Watering system*	Private	105	8	Ref	-	-
	Communal	417	20	0.6	0.2–1.4	0.256

Key: *CI* Confidence Interval, *COR* Crude odds ratio, *Ref* Reference, *PPR* Peste des Petits ruminants, *significant at alpha value less than 0.05

### Multivariable logistic regression analysis

In the multivariable logistic regression analysis, flock size, co-rearing with sheep, grazing location, and age were significantly associated with PPRV seropositivity **(**Table 4**).**

**Table 4:** Multivariable logistic regression analysis in Animal-level Seroprevalence of PPRV antibodies and associated risk factors in goats at Mandhera district of Sahil region, Somaliland, 2023 (*n* = 522)

Variables	Category	Number. Examined	No. seropositive	AOR	95% CI	*P*-value
*Animal source*	Born in	404	19	Ref	-	-
	Purchased	118	9	3.5	0.7–18.2	0.126
*Flock size*	Small(< 45)	117	9	Ref	-	-
	Medium45-100	156	3	5.2	1.5–14.3	0.003*
	Large > 100	249	16	16	1.0–33.5	0.061
*Raising together with sheep*	No	78	6	Ref	-	-
	Yes	444	22	4.2	1.7–15.7	0.047*
*Grazing place*	Plain	105	9	Ref	-	-
	Mixed	78	5	0.005	0.00-0.1	0.004*
	Mountainous	339	14	0.6	0.2–1.8	0.413
*Age*	> 0.5 ≤ 1year	50	7	Ref	-	-
	1-3years	306	14	0.2	0.07–0.6	0.006*
	> 3years	166	7	0.1	0.03–0.4	0.001*
*New animal introduction* *Management system*	No	391	18	Ref	-	-
	Yes	131	10	2.5	0.5–11.3	0.205
	Semi-intensive	104	7	Ref	-	-
	Extensive	418	21	0.1	0.01–2.1	0.173

Key: *AOR* Adjusted odds ratio, *CI* Confidence Interval, *Ref* Reference, *PPR* Peste des Petits ruminants, *significant at alpha value less than 0.05

Hypothesized risk factors for PPRV occurrence were initially analyzed using univariable logistic regression. Variables such as “shifting animals” and “production system” were removed from the final model due to collinearity **(**Table 3). However, flock size, co-rearing with sheep, grazing place, and age were statistically significantly associated with PPR seropositivity in the multivariable logistic regression analysis **(**Table 4**).**

## Discussion

The overall seroprevalence of 5.3% observed in this study confirms the endemic circulation of PPRV in the Mandhera district. This finding is consistent with the PPRV seroprevalence of 6.5% that was previously reported in Somaliland [10] and rates from 0% to 52.5% found in parts of Ethiopia [11]. However, it is notably lower than the seroprevalence values reported in southern Somalia (37.6%) or countries like Uganda (42%) and Saudi Arabia (55.9%) [10, 14, 15]. These disparities are likely due to differences in livestock density, mobility patterns, and the timing of sampling relative to local outbreaks.

Flock size was identified as a major risk factor with medium-sized flocks being five times more likely to be seropositive than small flocks. This observation is supported by studies in Ethiopia and Jordan, which suggest that higher animal density within a flock facilitates contact and virus transmission [16, 17]. While large flocks showed a high odds ratio (16.0), the result was not statistically significant, possibly due to the smaller number of very large herds sampled.

The significant association between co-rearing goats with sheep and higher seropositivity (AOR = 4.2) highlights the role of inter-species transmission. This findings align with observations in the Oromia region of Ethiopia, where mixed-species grazing was linked to higher virus circulation [3]. This increased circulation may be attributed to the fact that sheep often exhibit milder clinical signs and can thus serve as a subclinical source of infection for goats [18].

The study found that goats grazing in mixed grasslands had lower odds of seropositivity compared to those on plain communal grasslands. Plain grasslands in this region often involve higher concentrations of animals from different herds at watering points, increasing the risk of aerosol and fomite-mediated transmission. Conversely, mixed or mountainous terrains may naturally limit the frequency of contact between large herds.

Stratified by age analysis showed that younger goats (6–11 months) were more likely to be seropositive than older groups. This may indicate a recent “window” of infection following the waning of maternal antibodies. While some studies suggest seroprevalence increases with age due to cumulative exposure [11], our findings suggest that in endemic settings with sporadic virus circulation, younger animals may represent the most recent wave of infection.

A limitation of this study is its cross-sectional nature, which provides a snapshot in time and cannot establish seasonal trends. Additionally, while we focused on unvaccinated animals, the possibility of unrecorded vaccinations or movements of vaccinated animals into the district cannot be entirely excluded (recall bias).

## Conclusions and recommendations

This study establishes that PPRV is circulating among unvaccinated goats in the Mandhera district, Somaliland at a low but significant level. Key risk factors include medium flock sizes, co-rearing with sheep, and grazing on plain grasslands.

To support the global goal of PPR eradication by 2030, several strategic interventions are recommended based on these findings. First, vaccination efforts should be prioritized for large and mixed-species flocks, particularly in high-density grazing areas where the risk of transmission is greatest. Furthermore, the implementation of continuous longitudinal surveillance is essential to accurately identify the specific PPRV lineages circulating within the Sahil region. Finally, community education initiatives should be developed to inform pastoralists of the risks associated with mixing species and to emphasize the critical importance of reporting suspected outbreaks to veterinary authorities.

## Supplementary Information

Supplementary Material 1

Supplementary Material 2

Supplementary Material 3

## Data Availability

The datasets used and/or analyzed during the current study are available from the corresponding author upon reasonable request.
