# Supplementary material for: Seroprevalence and potential risk factors of peste des petits ruminants in goats in Mandhera District, Sahil Region, Somaliland
Source: BMC Vet Res. 2026 Feb 27;22:200. doi: 10.1186/s12917-026-05359-1 (PMC13049712; doi:10.1186/s12917-026-05359-1)
Supplement: Supplementary file 1 — Supplementary Material 1 [file 12917_2026_5359_MOESM1_ESM.pdf]

JAMUURIYADA SOMALILAND
WASAARADA X.XOOLAHA &
HORUMARINTA KALUUMAYSIGA

REPUBLIC OF SOMALILAND
MINISTRY OF LIVESTOCK
AND FISHERY DEVELOPMENT

Director General

Ref: W.X.X.H.K./AG/05/1070/2023 Date:30/08/2023

To: Department of Animal Science, College of Agricultural Science, Arba Minch University, **SNNP Ethiopia**

Title of the research proposal: The prevalence and potential risk factors of Peste des Petits Ruminants in Somali breed of goat in Mandhera district, Sahil region, Somaliland

Ethical considerations: On behalf of the Ministry of Livestock and Fishery Development, the above indicated research title of the Master program applicant **Abdirahman Saed Abdi (DVM, MSc fellow)** for target animal species; **Somali breed of goat**, in Study area; **Mandhera district, Sahil region, Somaliland** is acceptable from ethical perspective, relevance data collection, originality and technical competence points of view.
I hereby give ethical approval in respect of the undertakings contained in the above-mentioned title of the research proposal. Should any other instruments, test and laboratory be used, these require separate authorization. The Researcher may therefore commence with the research as from the date of this letter, using the reference number indicated above.

WASAARADDA XANNAANADA XOOLAHA & HORUMARINTA KALLUUMAYSIGA Director General Agaasimaha Guud MINISTRY OF LIVESTOCK & FISHERY DEVELOPMENT

Dr. Ahmed Ali Mah
Director General,
Ministry of Livestock and Fishery development

ahmed.maah@gmail.com Email:dg.moldf@sldgov.org
