# Supplementary material for: Seroprevalence and potential risk factors of peste des petits ruminants in goats in Mandhera District, Sahil Region, Somaliland
Source: BMC Vet Res. 2026 Feb 27;22:200. doi: 10.1186/s12917-026-05359-1 (PMC13049712; doi:10.1186/s12917-026-05359-1)
Supplement: Supplementary file 2 — Supplementary Material 2 [file 12917_2026_5359_MOESM2_ESM.docx]

| Date of sampling |  | | | Herd number | | | |  | | Name of owner | |  | | | | Name of village | |  | |
| --- | --- | --- | --- | --- | --- | --- | --- | --- | --- | --- | --- | --- | --- | --- | --- | --- | --- | --- | --- |
| Sample number | Sex (tick box) | | Dentition (pairs permanents incisors)  (tick box) | | | | | | | S-ELISA +ve or -ve | | | Clinical signs (describe) | Vaccination | | | | | |
|  | M | F | 0 | | 1 | 2 | 3 | 4 | 4* | +ve | -ve | |  | PPR | Yes | | No | |  |
|  |  |  |  | |  |  |  |  |  |  |  | |  |  |  | |  | |  |
|  |  |  |  | |  |  |  |  |  |  |  | |  |  |  | |  | |  |
|  |  |  |  | |  |  |  |  |  |  |  | |  |  |  | |  | |  |
|  |  |  |  | |  |  |  |  |  |  |  | |  |  |  | |  | |  |
|  |  |  |  | |  |  |  |  |  |  |  | |  |  |  | |  | |  |
|  |  |  |  | |  |  |  |  |  |  |  | |  |  |  | |  | |  |
|  |  |  |  | |  |  |  |  |  |  |  | |  |  |  | |  | |  |
|  |  |  |  | |  |  |  |  |  |  |  | |  |  |  | |  | |  |
|  |  |  |  | |  |  |  |  |  |  |  | |  |  |  | |  | |  |
|  |  |  |  | |  |  |  |  |  |  |  | |  |  |  | |  | |  |
|  |  |  |  | |  |  |  |  |  |  |  | |  |  |  | |  | |  |
|  |  |  |  | |  |  |  |  |  |  |  | |  |  |  | |  | |  |
|  |  |  |  | |  |  |  |  |  |  |  | |  |  |  | |  | |  |
|  |  |  |  | |  |  |  |  |  |  |  | |  |  |  | |  | |  |
|  |  |  |  | |  |  |  |  |  |  |  | |  |  |  | |  | |  |
|  |  |  |  | |  |  |  |  |  |  |  | |  |  |  | |  | |  |
|  |  |  |  | |  |  |  |  |  |  |  | |  |  |  | |  | |  |
|  |  |  |  | |  |  |  |  |  |  |  | |  |  |  | |  | |  |
|  |  |  |  | |  |  |  |  |  |  |  | |  |  |  | |  | |  |

Appendix II: Questionnaire format for serum sampled goats ( Laboratory Protocol)
