# Supplementary material for: Seroprevalence and potential risk factors of peste des petits ruminants in goats in Mandhera District, Sahil Region, Somaliland
Source: BMC Vet Res. 2026 Feb 27;22:200. doi: 10.1186/s12917-026-05359-1 (PMC13049712; doi:10.1186/s12917-026-05359-1)
Supplement: Supplementary file 3 — Supplementary Material 3 [file 12917_2026_5359_MOESM3_ESM.docx]

**Appendix I: Questionnaire Format for PPR Risk Factor Investigation (Flock/Herd or Cluster)**

1. **Owner information**: date: __/____/2023

Address: _________________________ phone #: __________

1. **Area information:**

Region______________ district___________ village__________

Agro-ecology: lowland______ midland ______highland _________

1. **Animal information**
2. Flock size: small(<45)______ medium (45_100)_______large(>100) _____
3. Raising together with sheep: yes _______ no _______
4. Sex: male_____ female ______
5. Age: (6 months to 1year) _____________ (1__3 years) _____ >3years______
6. New animal introduction: yes________ no ______________
7. BCS: (1very,thin)_____(2thin)_____(3good)_____(4fat)_____(5obese)______
8. Breed of goat: ___________________________(if any, different)
9. **Production and Housing System:**
10. Animal source: Purchased __________ Born at home_____________________
11. Introduction of a new animal into the flock: yes ____________ no __________
12. Free movement of animal: yes ________________ no ______________
13. Wild life contact: yes _______________________ no ______________
14. Housing system: Fence stable _______ House Barn_________ other _________
15. Share a common house with others: yes ________________ no ______________
16. Production system: Sedentary _____ Agro-pastorals______ Pastorals_____
17. **Grazing and watering management:**
18. Management system: intensive ______semi-intensive _______extensive _______
19. Shifting of the animals from place to place for feed: yes ______ no _______
20. Share common grazing land: yes ________no __________
21. Grazing place: plain __________mixed ____________Mountainous _______
22. Watering system: private _______________ communal ____________
23. **Vaccination and treatment:**
24. Vaccination: Yes____ No _____
    1. (If yes what vaccine and when)______________________
25. Treatment: Yes ______ No_____
    1. (If yes what, when drug)________________________________
